# Clinicopathological characteristics and diagnosis of hepatic sinusoidal obstruction syndrome caused by Tusanqi – Case report and literature review

**DOI:** 10.1515/med-2023-0737

**Published:** 2023-06-12

**Authors:** Youwen Tan, Sainan Zheng

**Affiliations:** Department of Hepatology, Third Hospital of the Zhenjiang Affiliated Jiangsu University, No. 300, Daijiamen, Runzhou Distinct, Zhenjiang 212003, China

**Keywords:** hepatic sinusoidal obstruction syndrome, pyrrolizidine alkaloid, Tusanqi, drug-induced liver injury

## Abstract

Tusanqi-induced hepatic sinusoidal obstruction syndrome (HSOS) is caused by exposure to pyrrolizidine alkaloids (PAs) and manifests as abdominal distension, liver pain, ascites, jaundice, and hepatomegaly. Pathologically, hepatic congestion and sinusoidal occlusion are observed in HSOS. We summarized the clinical characteristics of 124 patients with HSOS caused by Tusanqi in China between 1980 and 2019, along with those of 831 patients from seven English case series. The main clinical manifestations of PA-HSOS included abdominal pain, ascites, and jaundice. Common imaging features included characteristic heterogeneous density, slender hepatic veins, and other nonspecific changes. The acute stage is primarily manifested as hepatic sinus congestion and necrosis. Meanwhile, the persistence of hepatic sinus congestion and the onset of perisinusoidal fibrosis were observed during the repair stage. Finally, the persistence of hepatic sinusoidal fibrosis and resultant central hepatic vein occlusion were observed in the chronic stage. The new Nanjing standard for PA-HSOS incorporates the history of PA consumption and imaging features and eliminates weight gain and the serum total bilirubin value. Preliminary clinical validation of the Nanjing standard for PA-HSOS diagnosis revealed a sensitivity and specificity of 95.35 and 100%, respectively.

## 1 Introduction

Herb and dietary supplement-induced liver injury (HILI) is the main cause of drug-induced liver injury (DILI) in China, with an incidence rate of 23.8 cases per 100,000 residents, which is higher than that in Western countries [1]. Diagnosing DILI, especially HILI, is challenging because of the lack of characteristic clinical features and specific tests for this condition. Among the different hepatotoxic herbs, the pyrrolizidine alkaloid (PA)-containing herb [2], Gynura japonica or Tusanqi, can cause abdominal distension, liver pain, ascites, jaundice, and hepatomegaly. Recent studies have suggested that the pathophysiologic mechanism of Tusanqi is related to that of hepatocellular carcinoma [3]. The symptoms observed are mainly because of sinus endothelial injury, congestion, and hepatic sinusoidal obstruction syndrome (HSOS). This review describes the clinical and pathological features of PA-HSOS and discusses its diagnosis based on case studies.

## 2 Epidemiology

Exposure to PAs has been identified as one of the two major causes of HSOS [1,4]. PAs are common plant toxins that are widely distributed in up to 13 distantly related angiosperm families in the plant kingdom, including approximately 3% of flowering plants [5]. Humans are exposed to PAs through direct consumption of PA-containing plants [6,7] used as herbal medicines, herbal teas, and dietary supplements or through the ingestion of PA-contaminated foods [8,9], such as milk, tea, and honey. The earliest case of PA-induced HSOS was reported in 1920 and was caused by the ingestion of PA-contaminated wheat [10]. Since then, over 15,000 PA poisoning cases have been recorded in several countries and regions, including Afghanistan, Britain, China, Germany, Hong Kong (China), India, Jamaica, South Africa, Switzerland, and the United States [11]. In China, the main cause of HSOS is the ingestion of PA-containing herbal medicine (Gynura japonica, Tusanqi), which accounts for 50–89% [12] of the reported HSOS cases [13–18]. Between 1980 and 2019, a total of 2,156 Tusanqi-induced HSOS cases were reported in China: (1) 91 Tusanqi-induced HSOS case reports, involving 124 patients; (2) 87 Tusanqi-induced HSOS case series, involving 1,645 patients; and (3) 24 case series of Tusanqi-related HSOS (caused by mixed etiologies, including Tusanqi), involving 387 patients [19].

## 3 Pathogenesis

**Figure 1:**
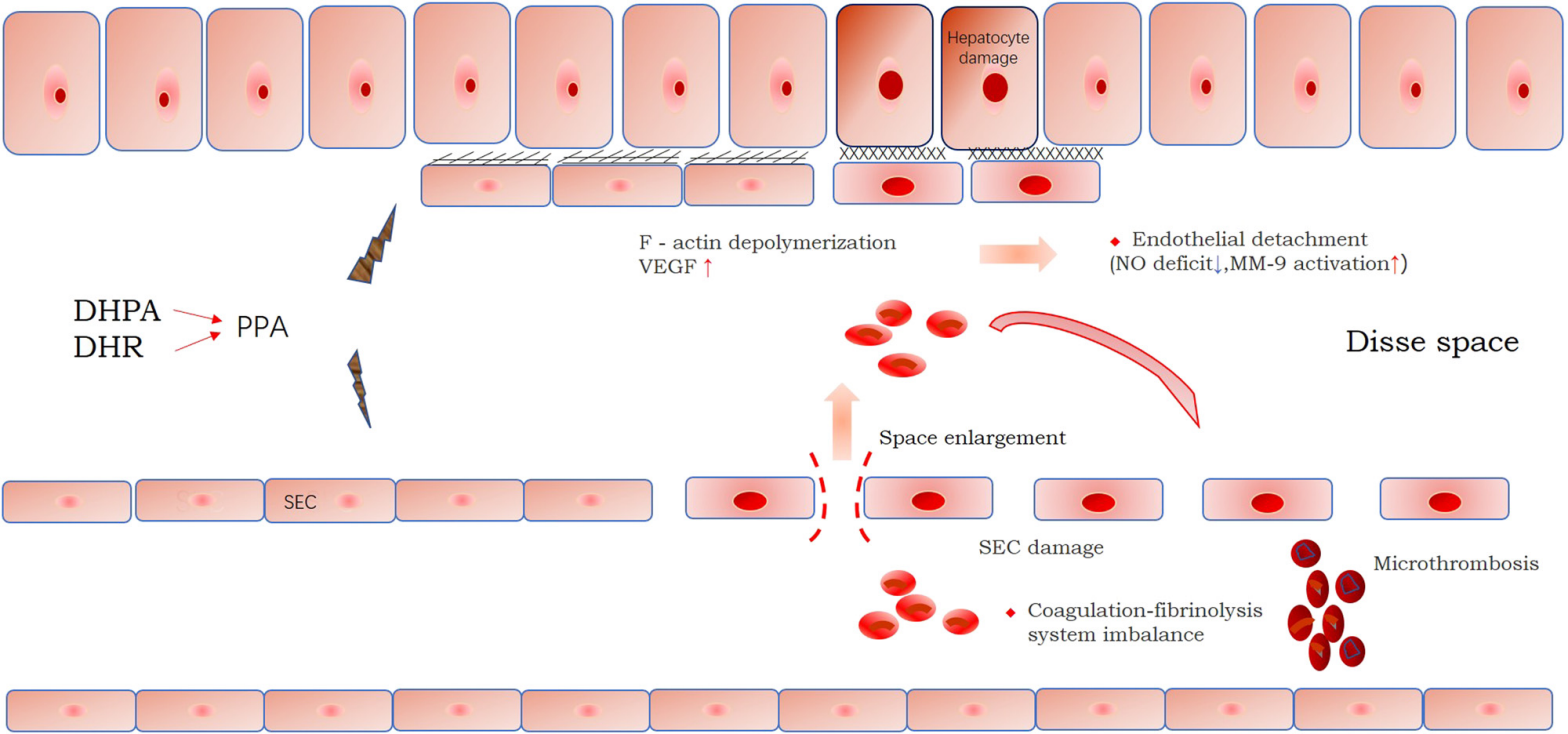
Pathogenesis of pyrrolizidine alkaloid-induced hepatic sinusoidal obstruction syndrome (PA-HSOS). DHR: dehydroretronecine, DHPA: dehydropyrrolizidine alkaloids, PPA: pyrrole–protein adducts, MMP-9: matrix metalloproteinase-9, VEGF: vascular endothelial growth factor, NO: nitric oxide.

PA is mainly composed of senilin and senilic acid and can be classified into two types: unsaturated and saturated PA. Saturated PA is generally considered to be non-hepatotoxic. However, unsaturated PA is genotoxic, carcinogenic, and hepatotoxic in humans and animals [20]. The prototype compound of unsaturated PA is less toxic as it is oxidized, nitroxidized, and demethylated to dehydropyrrolizidine alkaloids (DHPAs) by CYP3A (P450 cytochrome) in the liver and then hydrolyzed to dehydropyrrole and dehydroretronecine (DHR) [21,22], both of which bind to proteins to form pyrrole–protein adducts (PPAs) [23,24] that damage sinusoidal endothelial cells (SECs). SEC’s damage marks the beginning of PA-HSOS; thus, the protection of SECs can prevent the development of PA-HSOS [25,26]. The initial damage to SECs can be attributed to F-actin depolymerization caused by the covalent binding of PA-reactive metabolites to F-actin. Some studies have suggested that overexpression of thrombospondin 1 plays an important role in early SEC injury [27]. Since actin has a major role in maintaining the normal morphology of SECs, its depolymerization makes SECs more rounded and induces the release of matrix metalloproteinase-9 (MMP-9) [28,29]. Upregulation of MMP-9 leads to extracellular matrix (ECM) degradation in the space of Disse between the SECs and hepatocytes [30]. Morphological changes in endothelial cells and degradation of the ECM result in disruption of the liver sinusoidal barrier and efflux of red blood cells into the discharge space. Subsequent SEC necrosis, shedding, and obstruction of the hepatic sinusoids lead to PA-HSOS. In addition, the vascular endothelial growth factor (VEGF) upregulates the expression of MMP-9 by activating SECs [31]. The upregulation of MMP-9 is also associated with persistent extracellular regulated protein kinase 1/2 phosphorylation and decreased hepatic venous nitric oxide concentration [32]. The inhibition of MMP-9 production may be used as a target for the prevention and treatment of PA-HSOS. It has been reported that MMP-9 inhibitors can prevent PA-HSOS induced by monocrotaline in animal models [33]. In addition, distinct hepatic endothelial cell populations (Kupffer cells, SECs, and hepatic stellate cells) are required for complete microvascular function. SECs are unique endothelial cells that function as scavenger cells, removing circulating waste molecules and antigens. Under the synergistic effect of Kupffer and hepatic dendritic cells, they may also be involved in the immune regulatory function of the liver [34]. When SECs are damaged, their associated functions are no longer performed, contributing to the pathogenesis of PA-HSOS. After unsaturated PAs enter the human body, they can form electrophilic DHPA and DHR mediated by CYP3A. These proteins bind to form PPAs, which then damage SECs, thereby initiating PA-HSOS. Under the action of PA-reactive metabolites, F-actin depolymerization and MMP-9 upregulation damage the SECs shed and block hepatic sinusoids, exacerbating PA-HSOS. SEC’s dysfunction also further aggravates PA-HSOS. Besides, PAs damage hepatocytes through cytoskeleton necrosis and collapse [35] and induction of apoptosis [36] contributing to the development of PA-HSOS as shown in Figure 1.

## 4 Clinical manifestations

We summarized the clinical characteristics of 124 patients with HSOS caused by Tusanqi in China between 1980 and 2019 [19], along with those of 831 patients from seven English case series [12,37–42] (Table 1). The data of 124 patients from 74 institutions across 20 provinces and regions in China were analyzed. The clinical features of patients with Tusanqi-induced HSOS ranged from asymptomatic to severe. Symptoms usually appeared a few days to several years after ingestion of Tusanqi. The most common clinical features were typical HSOS symptoms, including ascites, hepatomegaly, and jaundice. Serum levels of alanine aminotransferase (ALT), aspartate aminotransferase (AST), alkaline phosphatase (ALP), gamma-glutamyl transpeptidase (GGT), and total bilirubin (TBIL) were measured. Some patients presented with mildly decreased serum albumin (ALB) levels and a prolonged prothrombin time (PT). Most patients underwent ultrasonography and computed tomography (CT) to diagnose HSOS. Meanwhile, liver biopsies were not universally performed.

**Table 1:** Demographic information, clinical manifestation, and laboratory tests of PA-induced HSOS

Reference	Zhu et al. [19]	Wang et al. [37]	Zhuge et al. [51]	Song et al. [38]	Wang et al. [39]	Zhang et al. [40]	Peng et al. [41]	Kan et al. [42]
Number (*n*)	124	117	108	116	84	86	249	71
Gender (male/female)	68/56	78/39	66/42	78/38	45/39	49/37	148/101	48/23
Age (year)	54.16 ± 21.52	63 (52.5–69)1	61 (55–69)1	56.92 ± 12.392	52.97 ± 15.282	65 (60–75)	64	56.48 ± 11.84
**Clinical manifestation**								
Ascites	100% (124/124)	99.1% (116/117)	98% (50/51)	100% (79/79)	100% (83/83)	100% (86/86)	100% (249/249)	100% (71/71)
Hepatomegaly	64.8%(79/124)	70% (82/117)	92% (47/51)	75.95% (60/79)	65.1% (54/83)		88.4% (220/249	78.87 (56/71)
Abdominal distention		98.3% (115/117)	99.1% (107/108)	98.3% (113/115)			99.2% (247/249)	100% (71/71)
Jaundice	57.4% (70/124)		39.8% (43/108)	52.9% (54/102)	57.8% (48/83)		40.1% (100/249)	
Right upper quadrant pain		19.7% (23/117)		36.4% (40/110)			34.1% (85/249)	61.97% (44/71)
Edema	41.0%(50/124)	39.3% (46/117)		39.5% (43/109)	37.3% (31/83)			36.62% (26/71)
Weight gain		18% (21/117)		15.5% (16/103)				11.59% (8/69)
**Laboratory tests**								
Variable	Mean ± SD	Median (25–75th percentiles)	Median (25–75th percentiles)	Mean ± SD	Mean ± SD	Median (25–75th percentiles)	Mean ± SD	Mean ± SD
ALT (U/L)	236.59 ± 385.08	49 (25.0–152.5)	52.6 (26.8–125)	134.50 ± 154.89	216.83 ± 235.78	40.9 (25.98–70.88)	132.09 ± 181.72	132.24 ± 155.83
AST (U/L)	222.15 ± 329.27	75 (39.0–158.5)	69.6 (42.4–115)	146.31 ± 156.30	221.15 ± 221.70	56.7 (38.25–91)	129.04 ± 184.08	148.00 ± 161.18
ALP (U/L)	180.50 ± 112.19	122 (86.8–191.3)	130 (105–178)	170 ± 106.89	183.48 ± 59.96		166.66 ± 80.49	177.91 ± 119.59
γ-GT (U/L)	131.96 ± 138.93	100.7 (61.8–164.8)	120 (72.4–170)	160.52 ± 114.56	155.70 ± 99.45			172.27 ± 128.21
TB (μmol/L)	64.72 ± 91.08	33.3 (19.7–47.0)	39.7 (28.3–62.4)	65.07 ± 78.83	52.96 ± 45.95	33.7 (22.38–46.2)	39.94 ± 28.87	69.81 ± 69.16
ALB (g/L)	32.25 ± 6.31	30.6 (27.7–33.6)	32.1 (29.8–34.7)	30.71 ± 5.50	32.02 ± 4.51		33.15 ± 4.31	30.76 ± 6.05
PT (s)	17.93 ± 7.51	14.8 (12.7–17.0)	15.1 (14.2–16.7)	17.43 ± 2.64	18.08 ± 4.12	14.55 (13–16.93)	16.01 ± 3.97	17.58 ± 2.87
PLT (109/L)		113 (78–153)	95 (74–134)	114.06 ± 63.48		99.5 (72.5–133.25)	115.32 ± 49.01	117.67 ± 64.60

ALT: alanine aminotransferase; AST: aspartate aminotransferase; ALP: alkaline phosphatase; γ-GT: γ-glutamyl transpeptidase; TB: total bilirubin; ALB: albumin; PT: prothrombin time; PLT: platelet.

## 5 Imaging features

Imaging features establish the diagnosis of PA-HSOS and are thus necessary for the examination of patients with this condition. Typical two-dimensional ultrasonography findings of PA-HSOS included [17]: diffuse hepatomegaly; hepatic parenchyma echogenicity showing thickening, increased density, and uneven distribution, with “patch-like” areas of reduced echogenicity along the hepatic veins; and ascites. Color Doppler ultrasonography showed normal portal and splenic vein diameters and reduced blood flow (<25 cm/s) [43]. Contrast-enhanced ultrasonography showed uneven enhancement during the arterial phase and slow filling of the portal vein and hepatic artery, in addition to prolonged arterial–hepatic vein transit time [43].

Typical CT findings included the following [42,44]: (1) diffuse hepatomegaly and non-uniformly reduced liver parenchyma density were observed on the plain scan; (2) liver parenchyma during the venous and equilibrium phases was characterized by a “map-like appearance,” “mottling-like” uneven enhancement, and low-density edema around the portal vein called the “halo sign”; (3) the caudate and left lateral lobes of the liver were slightly affected, liver parenchyma around the hepatic vein showed a high degree of enhancement, exhibiting the characteristic “clover sign,” lumen of the hepatic vein was narrow or unclear, and hepatic segment of the inferior vena cava was compressed and thinned out; and (4) extrahepatic signs, such as ascites, pleural effusion, gallbladder wall edema, and gastrointestinal wall edema, were also seen in combination. Patients in the acute stage had less complications, such as splenomegaly and esophagogastric varices, than those in later stages.

Typical magnetic resonance imaging (MRI) findings included [45] an enlarged liver and massive ascites on the plain scan, uneven liver signals, and slender or unclear hepatic veins. T2-weighted imaging also showed patchy high signals forming a “cloud” shape. An MRI dynamic enhanced scan showed uneven enhancement during the arteriovenous phase, forming a “piebald” shape, and enhancement was more evident during the delayed phase.

## 6 Clinical staging

Based on their clinical symptoms, the disease was divided into acute, subacute, and chronic stages. The staging criteria were as follows: (1) In the acute stage, early manifestations included weight gain, hepatomegaly, tenderness, and abnormal liver function. Patients also manifested abdominal distension, ascites, and jaundice. (2) In the subacute stage, persistent hepatomegaly, recurrent ascites, and liver dysfunction due to mild-to-severe or acute injury were observed. (3) In the chronic stage, the disease involved progression to liver cirrhosis, occurrence of hypersplenism, development of esophagogastric varices, and persistence of ascites.

## 7 Histopathological findings

A systematic description of pathological changes in the liver in PA-HSOS is lacking. One reason is that PA-HSOS has a high risk of puncture during the acute stage, and coagulation parameters and degree of ascites affect the decision to perform a liver biopsy. Although percutaneous or transjugular liver biopsy can reduce the risk of liver hemorrhage, technical difficulties preclude its use as a routine procedure. Besides, the diagnosis of PA-HSOS does not depend on liver pathology results. A study of 16 patients from a single center in China described pathological changes in the liver caused by PA-HSOS [46].

In the early stage, hematoxylin–eosin (HE) staining showed that hepatic sinusoids were dilated and congested at the center of the hepatic lobule. CD31 immunohistochemical staining showed that sinusoidal endothelium was incomplete, and reticular fiber staining showed numerous slender reticular fibers interspersed between red blood cells. Meanwhile, hepatic sinusoids without reticular fibers were narrowly compressed. In mild cases, a greater number of hepatic plates and sinusoids remained. In cases of severe hemorrhage, sinusoidal endothelium was completely destroyed, perisinusoidal space and sinusoids were not easily distinguished, and many hepatic sinusoids were interspersed with reticular fibers. Red blood cells obstructed the sinus cavity, most of the hepatic plate in the central lobular zone was necrotic and disappeared, and the hemorrhage fused into a sheet. In severe cases, two zones or even one may be involved. CD34 staining showed that the central vein endothelium was either partially or completely lost. Subendothelial hemorrhage, edema, and many red blood cells were also observed. The lumen of the blood vessels was either narrow or occluded.

In the middle stage, erythrocytes in the hemorrhagic zone were lysed, reticular fibers were collapsed and dense, collagen fibers were gradually deposited, lacunae with blood flow were congested and expanded, and some lacunae were covered with sinus endothelial cells (CD31 positive, CD34 negative). A portion of the ischemic liver plate gradually atrophied and underwent necrosis; thus, blood supply remained sufficient. These findings were observed in adjacent rows. Some hepatic sinusoids around the bleeding zone underwent compensatory dilatation and lacunar enlargement. Red blood cells within the endothelium of the central vein disappeared, and the subendothelial filling was massive. Reticular fibers progressed from edematous stenosis to fibrous stenosis. Although most of the lumen was completely occluded, the growth of a few small blood vessels was observed.

In the late stage, a large amount of collagen was deposited into the hemorrhagic zone. The remaining liver plate was gradually repaired by widening and regeneration in zones of light hemorrhage. In severe cases, large areas of fibrous scar form and can be seen lining the vascular endothelium (CD34 positive) of most dilated cavities. Hepatocytes in the marginal zone regenerated in adjacent rows and were gradually incorporated into the fibrous septum. The subendothelial collagen in the central vein became dense, and the lumen recanalized and gradually enlarged.

Counting and staging of hemorrhagic zones showed that the same patient can simultaneously have early, middle, and late hemorrhagic zones and that the changes in hepatic sinusoids and central veins are synchronized. Patients with a high proportion of fresh hemorrhagic lesions have a relatively short disease course. In contrast, fibrotic lesions are characterized by late bleeding; thus, patients with a high proportion of fibrosis had a relatively long disease duration.

Findings also varied depending on the degree of lobular hemorrhagic injury. In cases of mild injury, central lobular atrophy and narrowing of the hepatic plate were observed, and hepatic plates were continuous or mostly discontinuous. In moderate injury, central lobular atrophy was observed, and the hepatic plate disappeared but zone 2 expansion did not occur. In severe cases, the hepatic plate atrophied, the central lobular band disappeared, and expansion to zone 2 or even zone 1 occurred.

## 8 Case analysis

### 8.1 Case 1

**Figure 2:**
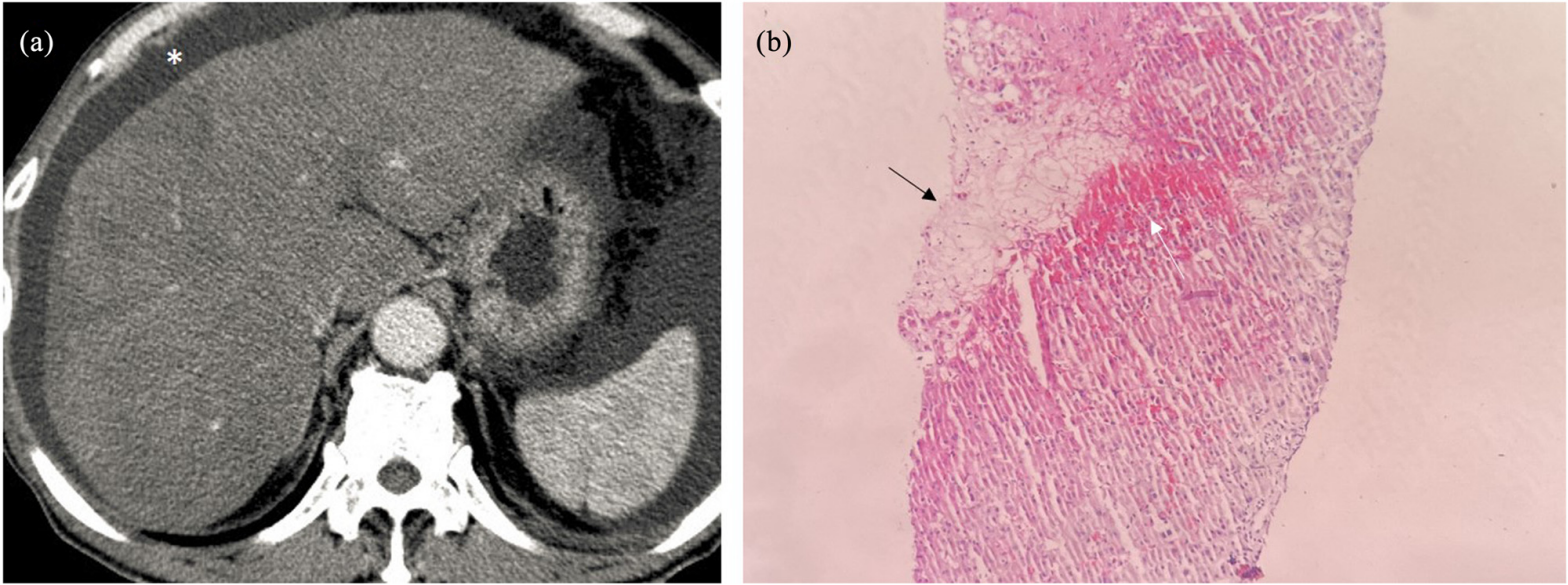
CT and pathological features in case 1. (a) CT scan shows diffuse hepatomegaly, heterogeneous density of the liver parenchyma, unclear delineation of the hepatic vein, and ascites (*). (b) Histopathology results reveal congestion, hemorrhage (white arrow), and coagulation-like necrosis (black arrow) in the central area of the liver (HE staining, 10 × 10).

A 52-year-old man ingested “Tusanqi powder” at a dose of 5 g one to two times daily because of an injury sustained from a fall. Eight days later, he developed fatigue, abdominal distension, and abdominal pain. His coagulation indices were as follows: PT: 19.0 s, prothrombin activity (PTA): 42%, and international normalized ratio (INR): 1.87. On biochemical testing, his results were as follows: estimated value of the glomerular filtration rate: 98.47 mL/min/1.73 m^2^, TBIL: 108.9 µmol/L, direct bilirubin (DBIL): 65.4 µmol/L, ALB: 26.0 g/L, globulin: 33.4 g/L, ALT: 645 U/L, AST: 477 U/L, ALP: 148 U/L, and GGT: 251 U/L. The CT scan showed that the density of the liver parenchyma was unevenly reduced. The liver parenchyma in the arterial and venous phases showed a characteristic “map-like” appearance and uneven enhancement, and the hepatic veins were thin and unclear. Enlarged lymph nodes in the porta hepatis were observed. A liver biopsy was performed on the 10th day of the disease. Early pathological changes were observed in the specimen of the patient who underwent liver biopsy during the acute stage. Hepatocytes exhibited hemorrhagic necrosis and were fused into sheets. As hepatocytes separated, numerous red blood cells infiltrated the perisinusoidal space, and the perisinusoidal space expanded and compressed the hepatic sinus. The sinus cavity and perisinusoidal space fused and became indistinguishable, and the hepatic sinusoid appeared dilated and congested. Hemorrhagic necrosis was observed on HE staining (Figure 2). After the diagnosis of HSOS was established, treatment with low-molecular-weight heparin combined with warfarin was initiated; however, the condition of the patient continued to deteriorate. Complications, such as hepatic encephalopathy, hepatorenal syndrome, and gastrointestinal bleeding, occurred, and the patient died on the 30th day of the disease.

### 8.2 Case 2

**Figure 3:**
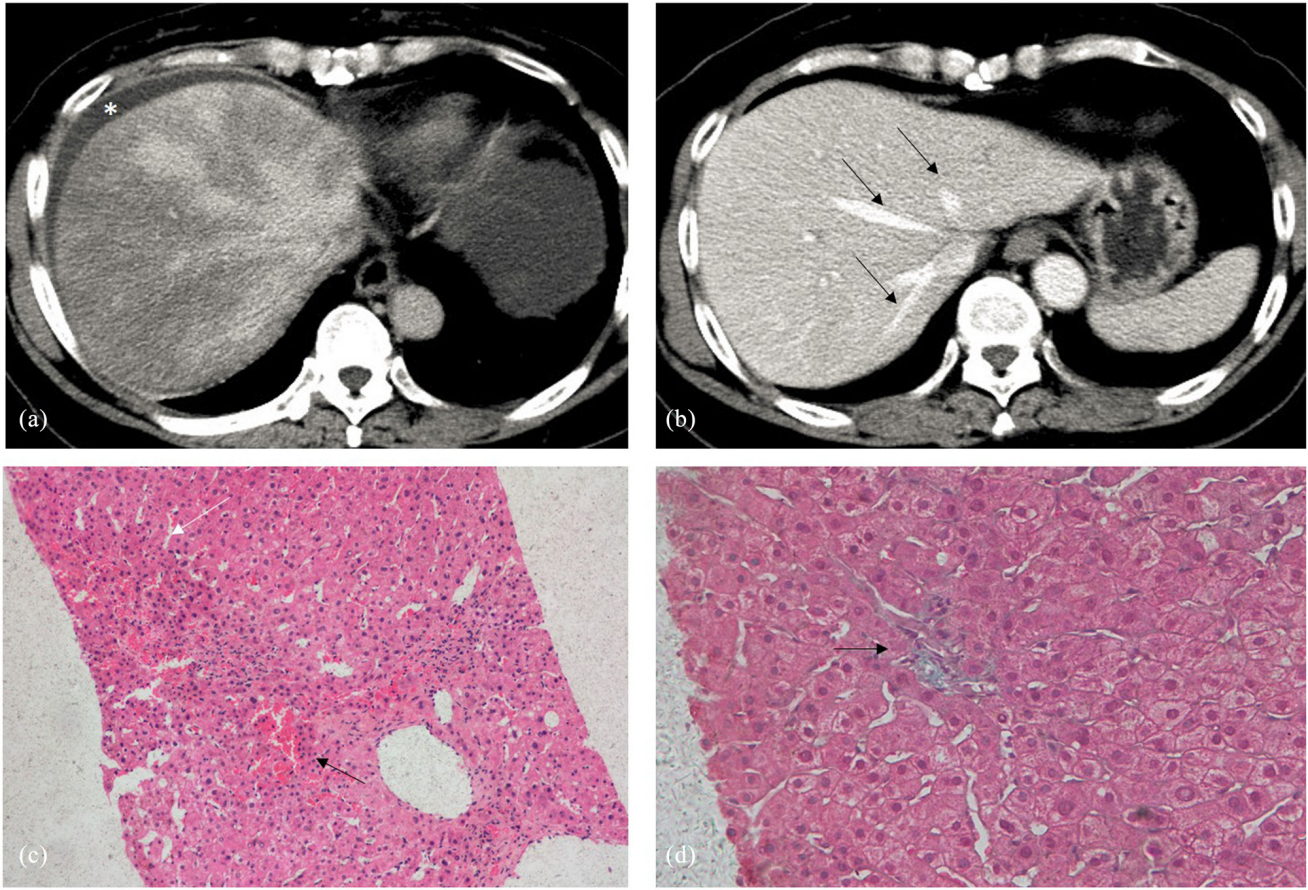
CT and pathological features in case 2. (a) CT scan shows heterogeneous density of the liver parenchyma, unclear clover-like enhancement of the hepatic vein, and ascites (*). (b) In the third month of the disease course, the CT scan shows homogenous density of the liver parenchyma and clearly exposed hepatic veins (black arrow). (c) Histopathology results show hepatic sinus congestion (black arrow) and inflammatory cell infiltration in a bridge-like pattern (black arrow) (HE staining, 10 × 10). (d) Mild fibrosis is seen around the sinus (black arrow) (Masson staining, 20 × 10).

A 49-year-old man ingested 3 g of “Tusanqi powder” three times daily for 2 months. Subsequently, the patient developed fatigue, abdominal distension, and abdominal pain. The laboratory findings were as follows: TBIL: 34.6 µmol/L, DBIL: 18.73 µmol/L, ALB: 34.2 g/L, ALT: 446 U/L, AST: 543 U/L, ALP: 116.0 U/L, GGT: 103 U/L, PT: 14.0 s, PTA: 77.8%, and INR: 1.13. CT showed diffuse hepatomegaly and unevenly reduced density of the liver parenchyma. The liver parenchyma during the arterial and venous phases showed a characteristic “map-like” appearance and uneven enhancement. The hepatic veins were unclear, and ascites were present. After admission, the patient was diagnosed with HSOS and treated with low-molecular-weight heparin and warfarin. After 1 month of treatment, the patient’s clinical symptoms disappeared and biochemical indices and imaging findings returned to normal. Warfarin was administered orally and monitored weekly to achieve an INR between 2.0 and 2.5. Three months after the patient was treated, a liver biopsy revealed that most liver tissues did not exhibit obvious inflammation and necrosis. Only a few areas still showed congestion in the hepatic sinus, with patchy and band-like inflammation and necrosis. Fibrotic embolism was observed in the central vein, and perisinusoidal fibrosis was also noted (Figure 3c and d).

### 8.3 Case 3

**Figure 4:**
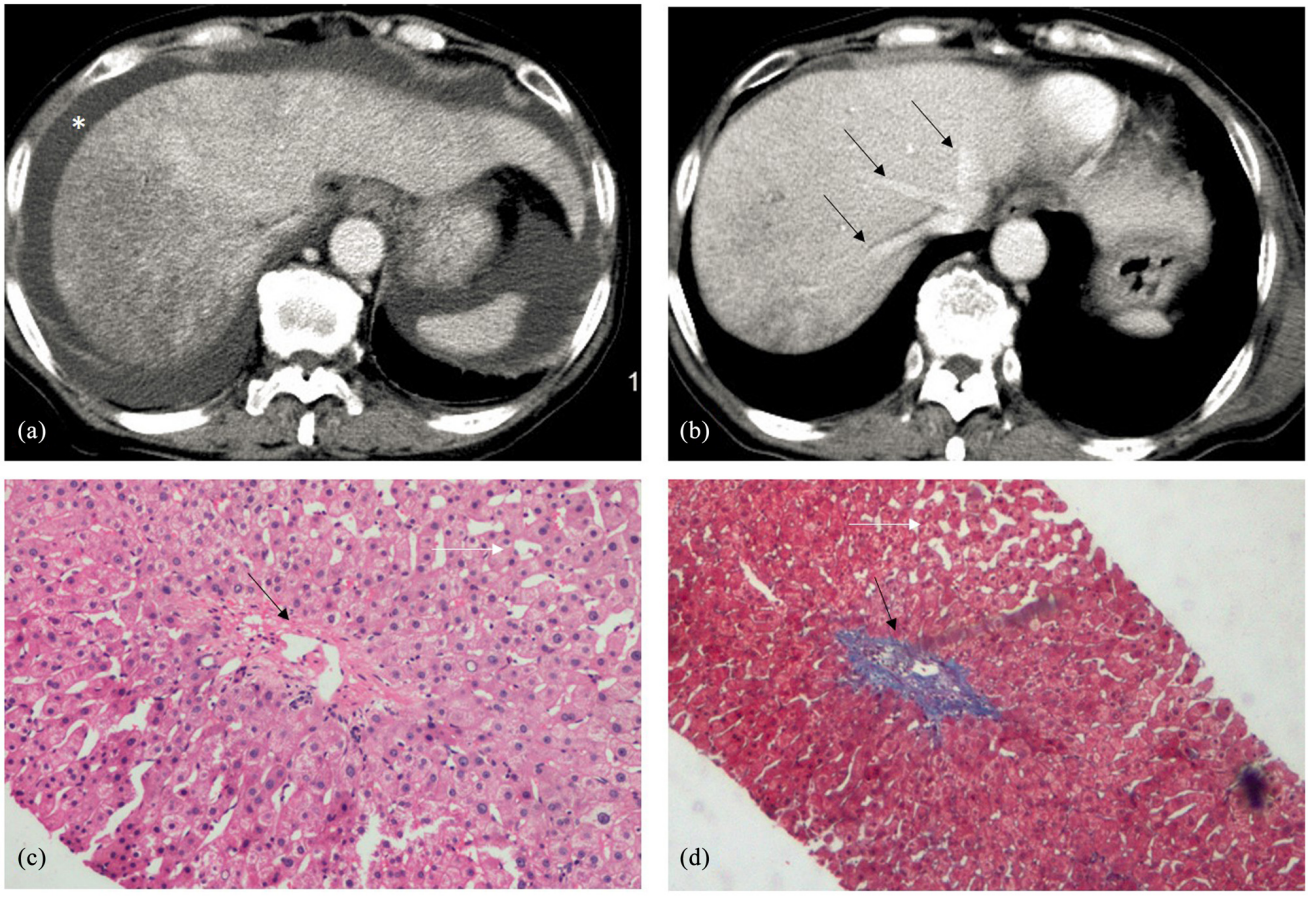
CT and pathological features in case 3. (a) CT scan shows diffuse hepatomegaly, heterogeneous density of the liver parenchyma, unclear delineation of the hepatic vein, and ascites (*). (b) In the eighth month of the disease course, the CT scan shows clearly exposed hepatic veins (black arrows) and homogenous density of most liver regions with the persistence of heterogeneity in a few remaining areas. (c) Histopathology results show central vein fibrosis (black arrow), absence of peripheral hepatocytes, infiltration of inflammatory cells, and hepatic sinusoidal dilatation (white arrows) (HE staining, 20 × 10). (d) Central venous occlusion (black arrow) and hepatic sinusoidal dilatation (white arrows) are observed (Masson staining, 10 × 10).

A 52-year-old woman intermittently ingested 3 g of “Tusanqi powder” one to two times a day. Fatigue, abdominal distension, and abdominal pain occurred after 15 days of using the drug. Her blood coagulation indices were as follows: PT: 15.0 s, PTA: 83%, and INR: 1.07. Biochemical test results were as follows: TBIL: 45.9 µmol/L, DBIL: 30.4 µmol/L, ALB: 31.0 g/L, ALT: 1142 U/L, AST: 1076 U/L, ALP: 118 U/L, and GGT: 296 U/L. Liver ultrasonography showed diffuse enlargement, liver parenchyma echogenicity with increased coarse density, uneven distribution, and visible “patch-like” areas of reduced echogenicity along the hepatic veins. A small amount of peritoneal effusion was also present. A CT scan showed an uneven reduction in liver parenchyma density, a characteristic “map-like” appearance of the liver parenchyma during the arterial and venous phases with uneven enhancement, and thin and unclear hepatic veins. Enlarged lymph nodes in the porta hepatis were also observed. After HSOS was diagnosed, treatment with low-molecular-weight heparin was initiated. After 30 days of treatment, the symptoms of the patient improved, and her biochemical indices and CT findings were close to normal (Figure 4a and b). The patient was readmitted for abnormal liver function in the 8th month of the disease and underwent a liver biopsy. Pathological features included mild inflammation in the central area, obvious dilatation of the hepatic sinus, mild fibrosis around the sinus, fibrosis deposition in the sinus, and an embolism in the central vein. Anticoagulant therapy with rivaroxaban tablets was then initiated for the patient (Figure 4c and d).

## 9 PA-HSOS diagnostic criteria: “Nanjing standard”

There are no unified criteria for diagnosing PA-HSOS, and its diagnosis is typically established using the modified Seattle and Baltimore criteria (Table 2). However, PA-HSOS and hematopoietic stem cell transplantation (HSCT)-HSOS have several differences in epidemiology, etiology, clinical features, and underlying diseases [12]. Thus, the use of the modified Seattle and Baltimore criteria for the diagnosis of PA-HSOS has significant limitations. In 2017, the “Expert Consensus Opinions on the Diagnosis and Treatment of PA-Related HSOS (2017, Nanjing)” proposed the “Nanjing standard” for the diagnosis of PA-HSOS (Table 2) [12], which preliminarily unified the criteria for PA-HSOS. In the Nanjing standard, the primary diagnostic criterion for HSOS is the use of PA-containing plants. In recent years, the incidence of PA-HSOS has increased. However, because the history of PA exposure is easy to miss, its incidence appears sporadic. The clinical manifestations of patients with HSOS are usually nonspecific; therefore, they are often misdiagnosed as acute or subacute severe hepatitis, decompensated liver cirrhosis, Budd–Chiari syndrome, or other diseases, affecting the timing of diagnosis and treatment of the disease and causing progression to severe or multiple organ failure with high mortality rates [47,48].

**Table 2:** Revised Seattle, Baltimore, and European Society for bone marrow transplantation (EBMT) diagnostic criteria for hepatic sinus obstruction syndrome

Standard	Definition
Revised Seattle standard	At least 2 of the following manifestations occurred within 20 days after HSCT: bilirubin >2 mg/dL, hepatomegaly with right upper abdominal pain, fluid retention, weight gain ≥ 2% of baseline weight
Baltimore standard	At least 2 of the following manifestations occurred within 21 days after HSCT: bilirubin >2 mg/dL, hepatomegaly with right upper abdominal pain, fluid retention, weight gain ≥ 5% of baseline weight
Nanjing standard	Have a clear history of taking plants containing PA, meet the following three items or be confirmed by pathology, and exclude liver injury caused by other known causes. ① Abdominal distension and/or liver pain, hepatomegaly, and ascites. ② Elevated serum total bilirubin or other abnormal liver function. ③ Typical enhanced CT or MRI findings

Note: HSCT: hematopoietic stem cell transplantation; PA: pyrrolizidine alkaloid; CT: computed tomography; MRI: magnetic resonance imaging.

## 10 Advantages of the “Nanjing standard”

The primary criterion in the “Nanjing standard” is a history of ingesting PA-containing plants. PA-HSOS is a DILI; therefore, obtaining a medication history is important. Domestic reports of HSOS are often higher than those of consumption of PA-containing plants. PA-containing plants are widely distributed. Approximately 3% of the flowering plants (more than 6,000 species) in the world contain PA, including comfrey (all genera), *Compositae* (ragwort and lycopi), and leguminous pig feces beans; however, Tusanqi is the most widely used PA-containing plant in China [47,49]. Many studies have reported the detection of PA in various foods, including honey, milk, salad, tea, and mutton [50].

Second, the “Nanjing standard” changed the criterion “serum bilirubin ≥ 34.2 μmol/L” to “elevated serum TBIL or other abnormal liver functions.” A recent review on PA-HSOS described four cohorts of 117, 108, 116, and 80 patients [22,37,39,51] with TBILs of 33.3 (19.7–47.0), 39.7 (28.3–62.4), 65.07 ± 78.83, and 52.96 ± 45.95 (μmol/L), respectively. Elevated bilirubin was not the main clinical feature of PA-HSOS, as approximately one-third of the TBIL levels of the patients were lower than 34.2 μmol/L. If the modified Seattle and Baltimore criteria were used to diagnose PA-HSOS, this finding may be missed. Therefore, the “Nanjing standard” no longer provides specific quantitative requirements for the elevation of serum TBIL.

Additionally, compared with the modified Seattle and Baltimore criteria, the “Nanjing standard” no longer incorporates weight gain as a criterion. Compared with patients with HSCT-HSOS whose body weights are regularly monitored during hospitalization, patients with PA-HSOS have sporadic presentations, and changes in their body weight are usually difficult to trace. The increase in their body weight is mainly due to the excess production of pleural fluid and ascites. In our summary of the clinical characteristics of PA-HSOS, we also found that only a few cohorts collected data on patient weight parameters (Table 1).

Importantly, imaging manifestations were added to the “Nanjing standard.” Patients with PA-HSOS share typical imaging characteristics that are valuable in the diagnosis of HSOS [52,53]. CT findings include ascites on the plain scan, uneven reduction of liver parenchyma density, characteristic “geographic” and “piebald” uneven enhancement, and varying degrees of abnormal liver patch enhancement associated with clinical severity. MRI findings include heterogeneous patchy enhancement of the liver parenchyma during the portal venous and delayed phases and poor filling of the hepatic lobe and segmental veins by the contrast medium [54]. A recent single-center retrospective study [42] validated the diagnostic value of contrast-enhanced CT among patients with PA-HSOS (*n* = 71), Budd–Chiari syndrome (*n* = 57), and liver cirrhosis (*n* = 165). In patients exhibiting patchy enhancement, the sensitivity, specificity, positive predictive value, negative predictive value, and accuracy for the diagnosis of PA-HSOS were 92.96, 92.79, 80.49, 97.63, and 91.83%, respectively. Contrast-enhanced CT showed ideal diagnostic performance and can therefore be used as an effective, simple, and noninvasive method for diagnosing PA-HSOS.

## 11 Verification of the “Nanjing standard”

The diagnostic performance of the “Nanjing Criteria” lacks systematic evaluation and verification. A recent clinical study [40] conducted preliminary clinical validation of the “Nanjing standard.” This study retrospectively analyzed consecutive patient data from multiple hospitals (ID: ChiCTR1900020784), including 86 patients with PA-HSOS and 327 patients with other liver diseases. The diagnostic performances of the Nanjing standard and simplified Nanjing standard were evaluated and validated. The sensitivity and specificity of the Nanjing standard for the diagnosis of PA-HSOS were 95.35% and 100%, respectively. However, this was a single-center retrospective study, and selection bias was present because of the small sample size and a small number of patients with certain diseases (such as acute liver injury and Budd–Chiari syndrome). Therefore, further research with multicenter and large-sample clinical trials is required to validate the “Nanjing standard” for the diagnosis of PA-HSOS.

In conclusion, PA-HSOS is a DILI that has been rising in recent years and has a high mortality rate, if not treated early. Early diagnosis is the key to initiating early treatment and obtaining a good prognosis. Understanding the key features of this disease enables early diagnosis. A clear history of PA use, characteristic clinical features, such as abdominal distension, abdominal pain, and ascites, that are significantly more severe than those in other liver diseases (such as viral hepatitis and alcoholic hepatitis), and characteristic imaging findings support the diagnosis of PA-HSOS. Although pathology is the gold standard for diagnosis, its invasiveness precludes its routine use. The “Nanjing standard” may also be valuable in the diagnosis of PA-HSOS.
